# Analysis of *Arabidopsis* floral transcriptome: detection of new florally expressed genes and expansion of Brassicaceae-specific gene families

**DOI:** 10.3389/fpls.2014.00802

**Published:** 2015-01-20

**Authors:** Liangsheng Zhang, Lei Wang, Yulin Yang, Jie Cui, Fang Chang, Yingxiang Wang, Hong Ma

**Affiliations:** ^1^Department of Pharmacy, Shanghai Tenth People's Hospital, School of Life Sciences and Technology, Tongji UniversityShanghai, China; ^2^Advanced Institute of Translational Medicine, Tongji UniversityShanghai, China; ^3^State Key Laboratory of Genetic Engineering and Collaborative Innovation Center for Genetics and Development, Ministry of Education Key Laboratory of Biodiversity Science and Ecological Engineering and Institute of Biodiversity Sciences, Institute of Plants Biology, Center for Evolutionary Biology, School of Life Sciences, Fudan UniversityShanghai, China; ^4^Institutes of Biomedical Sciences, Fudan UniversityShanghai, China

**Keywords:** *Arabidopsis thaliana*, RNA-Seq, differentially expressed genes, floral development, gene families

## Abstract

The flower is essential for sexual reproduction of flowering plants and has been extensively studied. However, it is still not clear how many genes are expressed in the flower. Here, we performed RNA-seq analysis as a highly sensitive approach to investigate the *Arabidopsis* floral transcriptome at three developmental stages. We provide evidence that at least 23, 961 genes are active in the *Arabidopsis* flower, including 8512 genes that have not been reported as florally expressed previously. We compared gene expression at different stages and found that many genes encoding transcription factors are preferentially expressed in early flower development. Other genes with expression at distinct developmental stages included *DUF577* in meiotic cells and *DUF220*, *DUF1216*, and *Oleosin* in stage 12 flowers. *DUF1216* and *DUF577* are Brassicaceae specific, and together with other families experienced expansion within the Brassicaceae lineage, suggesting novel/greater roles in Brassicaceae floral development than other plants. The large dataset from this study can serve as a resource for expression analysis of genes involved in flower development in *Arabidopsis* and for comparison with other species. Together, this work provides clues regarding molecular networks underlying flower development.

## Introduction

Flower is one of the most complex structures of the angiosperms (flowering plants), and is thought to make great contribution to sexual reproduction in either developmental or evolutionary aspects (Alvarez-Buylla et al., 2010). The basic floral architecture is highly conserved among the core eudicots, including *Arabidopsis thaliana*, which is an important model plant for studying flower development. Over the past three decades, extensive molecular genetic analyses have identified a large number of key floral regulators controlling flower development (O'Maoileidigh et al., 2014), making it one of the best-understood aspects of plant development. However, the present knowledge in understanding gene regulatory network in flower development is incomplete, such as information on genes with low expression levels.

Genome-wide approaches have become valuable tools in characterizing gene expression and in elucidating the genetic networks of flower development at a global level. In the past, large-scale analyses of transcript enrichment among *Arabidopsis* floral organs largely depends on hybridization, such as cDNA and oligonucleotide arrays (Hennig et al., 2004; Wellmer et al., 2004, 2006; Zhang et al., 2005; Alves-Ferreira et al., 2007; Benedito et al., 2008) and represents a major step in the spatial characterization of floral transcriptome, resulting in identifying many genes important for flower development (Alvarez-Buylla et al., 2010; Irish, 2010). However, array analyses and other hybridization-based approaches have several limitations, including knowledge of genes for probe design, non-specific hybridization, and difficulty in detecting low level expression (Marioni et al., 2008). On the other hand, more recently developed RNA sequencing (RNA-seq) technologies can overcome such limitations of hybridization-based approaches and other conventional large-scale gene expression analysis methods (Marioni et al., 2008; Xiong et al., 2010). It also has great sensitivity, allowing the detection of transcripts with lower expression levels, such as those of many transcription factors (Marioni et al., 2008; Chen et al., 2010). In the last few years, RNA-seq has been extensively applied in the characterization of transcriptome regarding developmental stage, organ, even specific cell types or single cell level, from yeast to human, including several plant species (Jiao et al., 2009; Zhang et al., 2010; Yang et al., 2011). To date, RNA-seq has been used for cell-specific analysis of actively translated mRNAs associated with polyribosomes in developing flowers, providing insights and resources to further study flower development (Jiao and Meyerowitz, 2010).

To further explore the *Arabidopsis* flower transcriptome, we employed RNA-seq for three developmental periods. We detected 8512 additional genes that are not present on previously used microarray experiments, and provide evidence that at least 23,961 genes are truly expressed in the *Arabidopsis* flower. We also identified differentially and specifically expressed genes and gene families during flower development.

## Materials and methods

### Sequencing datasets

The inflorescent meristem (IM), stage 1–9 flowers (F1–9) and stage 12 flowers (F12) samples for RNA-seq were collected in our lab, and the three samples were subjected to 50 bp single-end sequencing on a SOLiD 3 platform; details for the methods were recently described in a study for alternative splicing (Wang et al., 2014a). All sequenced short reads were submitted to NCBI Short Read Archive under accession number SRP035230. The datasets for seeding and stage 4 flowers were from previously studies, which generated 36-bp and 42-bp long reads, respectively, using the Illumina genome analyzer (Filichkin et al., 2010; Jiao and Meyerowitz, 2010). The meiocyte datasets were from our previous study, which included two runs (36 and 50 bp) using Life Technologies' SOLiD sequencing platform (Yang et al., 2011).

### Alignment of sequencing reads

Sequence reads from the three sample plus the three floral samples were mapped using PerM (Chen et al., 2009) to the *Arabidopsis* genome (release 9) from the *Arabidopsis* Information Resource (TAIR) database (TAIR9; www.arabidopsis.org) allowing 5, 4, and 3 mismatches per 50, 42, and 36-bp read, respectively.

### Digital gene expression and expression arrays

For the RNA-seq experiments, we used at least 10 reads mapped to a gene as the threshold for being expressed. The raw digital gene expression counts were normalized using the reads per kilo-base of mRNA length per million of mapped reads (RPKM) method. The equation was used:

$$RPKM=\frac{10^{9}\;*\;C}{N*L}$$

Where *C* is the uniquely mapped counts determined from mapping results, *L* is the length of the cDNA for the longest splice variant for a particular gene model and *N* is the total reads that were mapped to the genome. Log2-transformation of this normalized value was performed as in other analyses.

To test differential expression with mapping data DEGseq was used (Wang et al., 2010). Fisher's Exact Test (*P* < 0.01) method was selected. Microarray results were obtained from a previous study (Zhang et al., 2005). The Microarray experiments have a background value, which was 5 (log value of base 2) as previously described (Zhang et al., 2005) for the evaluation of “expressed” or “unexpressed” genes. Identification of differentially expressed genes according to the microarray data also used the Fisher's Exact Test method.

### Z-score

Calculation of the Z-score was based on the log2-transformed RPKM-normalized transcript levels as follows:

Z=(X−μ)/σ

*X* is the RPKM of a gene for a specific tissue/developmental stage. μ is the mean RPKM of a gene across all tissues/developmental stages and σ is the RPKM standard deviation of a gene across all tissues/developmental stages. All calculations and plotting were performed by Perl and excel, respectively.

### Gene family and functional annotation

The protein domain annotations were obtained from the Pfam database (http://pfam.sanger.ac.uk) (Punta et al., 2011). *Arabidopsis* protein sequences were then searched against protein family models in the Pfam-A database, resulting in 21102 *Arabidopsis* proteins identified as having at least one Pfam domain. Transcription factor family annotations were from The Database of *Arabidopsis* Transcription Factors (http://datf.cbi.pku.edu.cn/) (Guo et al., 2005), which contains 1922 transcription factors in *Arabidopsis*. Gene ontology (GO) enrichment analysis was performed with the agriGO browser (http://bioinfo.cau.edu.cn/agriGO/) (Du et al., 2010) using Singular Enrichment Analysis.

Multiple sequence alignment was performed in MUSCLE (http://www.drive5.com/muscle/) using the default parameters. Maximum likelihood (ML) trees were constructed by FastTree (www.microbesonline.org/fasttree) with the approximate likelihood ratio test method.

## Results and discussion

### Global gene expression of flower transcriptomes in *Arabidopsis*

To obtain more insights about the overall transcriptome landscape during flower development, we analyzed RNA-seq datasets of the *Arabidopsis* flower at three developmental stages recently generated in our laboratory; these datasets were analyzed for alternative splicing in a separate study (Wang et al., 2014a): inflorescent meristem (IM), stage 1–9 flowers (F1–9) and stage 12 flowers (F12), detecting 21,181 (IM), 22,137 (F1–9), and 22,827 (F12) reliably expressed genes (Table S1, Figure S1). A recent report summarized that a total of 126 *Arabidopsis* genes have been demonstrated genetically to have a role during flower development (Alvarez-Buylla et al., 2010), 122 of which were also detected as expressed in our dataset (Table S2), indicating that our data were very reliable, and can be used for further analysis. To compare gene expression during flower development, besides the three datasets described above, we also included data for *Arabidopsis* male meiocytes that we had generated previously (Yang et al., 2011), and two other public datasets of *Arabidopsis* seedlings and stage 4 flowers (Filichkin et al., 2010; Jiao and Meyerowitz, 2010), the latter of which were from isolated polysomic.

**Figure 1:**
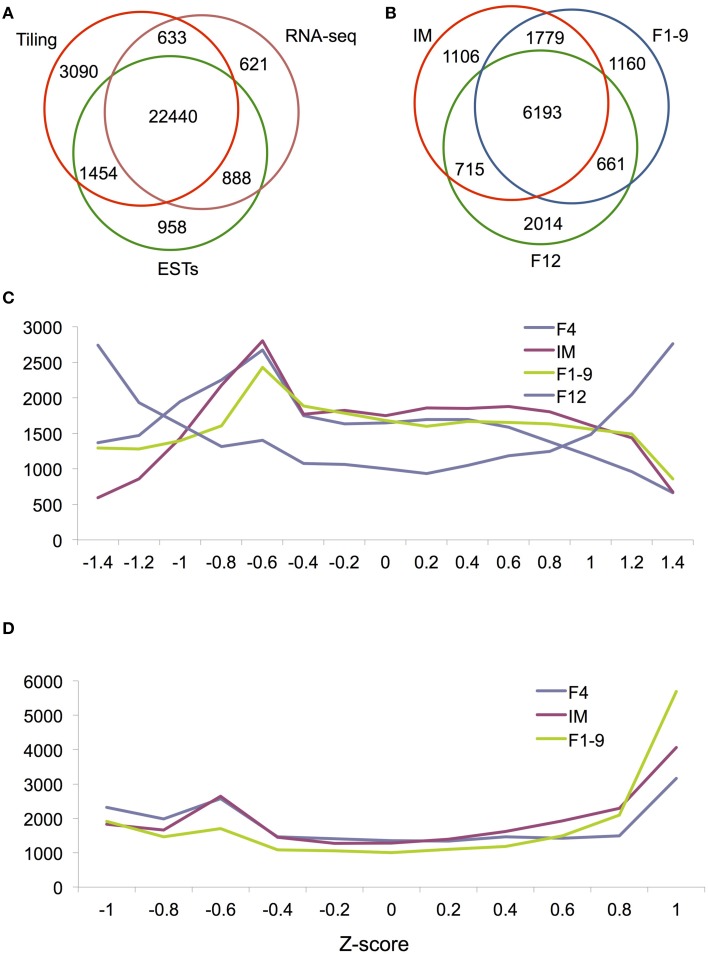
**Global gene expression during flower development. (A)** A Venn diagram showing the overlap in detected genes between three technologies: the tilling array, RNA-seq and ESTs. **(B)** A Venn diagram showing the overlap between IM, F1–9, and F12 for genes called as DEG by RNA-seq. Histograms of relative expression levels (measured by Z-scores) in four **(C)** and three **(D)** organs. For easy visualization, we plotted Z-score on the x-axis and gene numbers on the y-axis.

To further explore how many genes are truly expressed in *Arabidopsis* flower, we searched The *Arabidopsis* Information Resource (TAIR) database and obtained a total of 24,570 genes, which are supported by at least one EST. Then, we searched the present tiling array database to find available probes for 30,228 genes. 4734 genes were found to be tiling array-specific compared with ESTs and RNA-seq data. Among them, 2634 and 276 are transposons and pseudogenes, respectively. The other 1824 genes seem to be expressed at very low levels. The average value of the 4734 genes is 5.4, which is regarded as a threshold in this study for the evaluation of “expressed” or “unexpressed” genes. Based on this criterion, we believe that tiling array can detect at least 27, 617 genes. As described previously, RNA-seq detected 24,769 genes in flowers. Comparison of the detected genes among EST, tiling array and RNA-seq found that 22,440 genes were detected by three data sets and 1521 genes were detected by RNA-seq and either ESTs/tiling array (Figure 1A). The results suggest that at least 23,961 genes are reliably detected as expressed in the *Arabidopsis* flower. In addition, 621 genes were only detected by RNA-seq; most of these are low abundance genes that are nearly undetectable by arrays and the others are likely to be stage-specific genes.

Characterization of stage or cell-specific genes provides a foundation for unraveling their molecular mechanisms. Previous studies in multiple plants demonstrated that each stage or tissue has specific transcripts (Jiao et al., 2009; Jiao and Meyerowitz, 2010; Yang et al., 2011; Liu et al., 2014). To better establish the genome-wide gene expression pattern of flower development, we conducted a Z-score analysis to assess the extent of differential gene expression for florally expressed genes. Results showed that the Z-score distribution of gene expression in F12 was dramatically different from that for early flower development (F1–9, F4, and IM) (Figure 1C), suggesting that nearly mature flowers requires many more specifically or differentially expressed genes than early flowers. In contrast, Z-score distributions were very similar between F1–9, F4, and IM (Figure 1D), further supporting the idea that the developmental programs of these stage/organ are similar.

### Detection of expression of 8512 genes in the *Arabidopsis* flower not reported from microarray analysis

**Figure 2:**
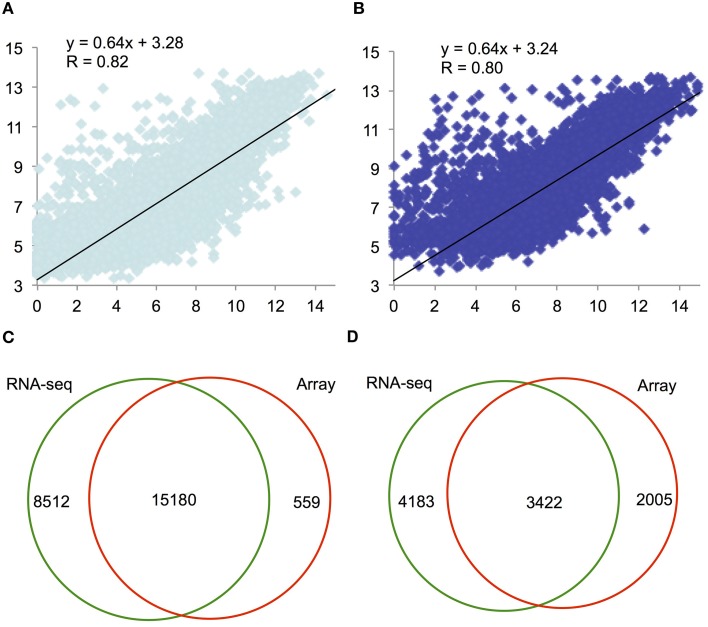
**A scatter plot of relative expression values obtained by RNA-seq and microarray for F1–9 and F12. (A)** Comparison of expression levels between RNA-seq and microarray for F1–9, RNA-seq and microarray; gene expression levels transformed with log2 were plotted. **(B)** Comparison of expression between RNA-seq and microarray for F12. **(C)** A Venn diagram illustrating genes detected by RNA-seq (left) and microarray (right) analyses. **(D)** A Venn diagram presenting the overlap of differentially expressed genes between F1–9 and F12 from RNA-seq (left) and from microarray analysis (right).

We first compared the F1–9 and F12 RNA-seq data with the Affymetrix ATH1 array data at similar stages (Zhang et al., 2005). We compared the number of sequencing reads mapped to each gene with the corresponding (normalized) absolute intensities from the array (Figures 2A,B), and found that the correlations between the two platforms were high, with Spearman correlation coefficients of 0.82 (F1–9; Figure 2A) and 0.80 (F12; Figure 2B). Thus, comparison of RNA-seq and microarray identified 15,180 overlapping genes with relatively high expression levels (Figure 2C), covering 96% of genes detected using microarray. In addition, our RNA-seq identified additional 8512 genes that were undetected by microarray (Zhang et al., 2005), whose expression levels were obviously lower; the average expression level is 56.12 (F1–9) and 57.82 (F12) RPKM (Figure S2b), compared to the average expression level about 250 for the 15,180 genes. Also, the curvature of the comparison toward the microarray axis suggested that microarray possibly underestimated the expression level of genes relative to RNA-seq (Figures 2A,B). Together, these results suggest that RNA-seq has a great advantage over microarray in detecting low-abundance transcripts, consistent with previous reports (Marioni et al., 2008; Yang et al., 2011).

To investigate further regarding the 8512 genes, we analyzed the enrichment of protein family (PFAM) domains (gene families) among these genes, and identified several enriched gene families that were not reported previously as enriched, including *F-box*, *NB-ARC*, *C1_3*, *PPR*, *LRR_1*, *Myb*, *bHLH*, and *AP2* gene families (Table 1). Previously, many *F-box* genes were reported as unexpressed or undetectable by microarray analysis (Schmid et al., 2005), further suggesting that microarray is not as sensitive as RNA-seq for detecting low-abundance transcripts. In addition, we also detected some enriched gene families that belongs to the highly expressed genes; for instance, Plant self-incompatibility response (SCRL) and S locus-related glycoprotein 1 binding pollen coat protein (SLR1-BP) are specifically enriched in F12 (Table 1 and Table S3), suggesting a potential role at this stage. In contrast, 559 genes detected by microarray were not found by RNA-seq, possibly due to difference in growth conditions.

**Table 1:** **Enriched protein family (Pfam) among 8512 genes that are undetected by microarray analysis**.

**Family**	**Total**	**RNA-seq**	**Percent**	**Family**	**Total**	**RNA-seq**	**Percent**
NB-ARC	167	108	0.65	bHLH	134	59	0.44
FBD	115	71	0.62	AP2	145	62	0.43
TIR	132	80	0.61	UDPGT	115	49	0.43
SCRL	25	14	0.56	peroxidase	82	33	0.40
U-box	61	31	0.51	Myb	256	103	0.40
MATH	60	30	0.50	Malectin_like	78	31	0.40
DUF26	97	47	0.48	LRR_1	391	154	0.39
Auxin	79	38	0.48	PMEI	122	46	0.38
C1_3	146	70	0.48	SLR1-BP	41	15	0.37
DUF295	78	36	0.46	p450	249	91	0.37
PPR_1	301	138	0.46	zf-rbx1	174	63	0.36
PPR	465	211	0.45	ABC_tran	117	42	0.36
NAC	113	51	0.45	Kelch_1	110	38	0.35
F-box	522	233	0.45	Ank_2	106	36	0.34
FBA_1	176	78	0.44	zf-C3HC4	306	96	0.31

We further employed a widely used Fisher's Exact Test method to identify differentially expressed genes (DEGs) between F1–9 and F12 in RNA-seq and microarray data. Altogether, 7605 and 5327 DEGs were identified in each dataset. Among them, 3422 genes were detected by both platforms (Figure 2D), 1272 and 84 DEGs were only detected by RNA-seq and microarray, respectively (Figure 2D), and consistent with the fact that RNA-seq is more sensitive for detection and comparison of gene expression. Taken together, these results indicate that deep sequencing can greatly increase the sensitivity of transcriptome analysis.

### Identification of stage-differentially expressed genes during flower development

Floral organ identity and cell fate determination are highly regulated by the temporal and spatial gene expression, with each organ or cell type having distinct transcriptomes (Jiao et al., 2009; Yang et al., 2011; Wang et al., 2014b). To investigate DEGs between one of the floral stages with seedlings, we compared the flower transcriptomes of IM, F1–9, or F12 with that of seedlings, and identified IM with 9793 DEGs, F1–9 with 9583 DEGs, and F12 with 9340 DEGs (Table 2). Furthermore, the intersection between these three sets contained 6193 genes (Figure 1B), indicating these three samples are quite similar regarding differentially gene expression compared with seedlings. GO annotation showed that these genes were enriched for categories such as “histone modification” and “methylation” (*p* = 3.2E-11 and 4.7E-9), suggesting these genes are involved in the establishment of transcription regulation during flower development. Likewise, 2014 genes were specifically expressed in F12 and showed significant enrichment for genes in reproduction (*p* = 2.2E-47), flower development (*p* = 2.9E-22) and post-embryonic development (*p* = 1.3E-194), which might suggest that genes expressed during gametophyte development can function in later stages.

**Table 2:** **Differentially expressed genes (DEGs) for each floral sample compared with seedling**.

	**Seedling**	**IM**	**F4**	**F1–9**	**F12**	**Meiocytes**
Seeding		9793	8703	9583	9340	4966
IM	6627		3747	6866	6943	3900
F4	5812	5354		6016	6460	3449
F1–9	6866	7228	4834		7697	3773
F12	5987	8960	6724	8109		3820
Meiocytes	5110	7167	6154	6628	6401	

To further examine the combined set (13,628 genes) of the above floral DEGs, we compared these genes with 4505 genes identified as potential targets of the SEP3 and/or AP1 proteins by ChIP-seq (Immink et al., 2009; Kaufmann et al., 2010). The results showed that 2506 genes overlapped between the floral DEGs and the SEP3/AP1 targets with significance (Fisher's test, *p* = 7.03e-08). It is likely that some of these genes are involved in the regulation of flower development, but the role of these genes in flower development needs to be determined using molecular genetic analyses.

We then analyzed the enrichment of protein domains as defined in the PFAM database, and found several enriched domains (*P* ≤ 0.01; Table 3), including ATPase, Helicase_C, DEAD box, WD40, SET, and PHD domains, suggesting that chromatin associated transcriptional regulation might be one of the major features underlying flower development. In addition, proteins with “UCH,” “hydrolase,” and “IQ” domains were also significantly over-represented, although their functions in flower development are largely unknown. Interestingly, we also identified “PPR,” “Mito_carr” and “Miro” domains as significantly enriched; members of these genes are involved in gene expression and other functions in mitochondria and plastid, suggesting that such organellar functions might be important for flower development.

**Table 3:** **Significance of enriched Pfam domains in differentially expressed genes during flower development**.

**Pfam domain**	**Total**	**Num.**	**Percent**	***P*-vaule**	**Pfam domain**	**Total**	**Num.**	**Percent**	***P*-vaule**
Helicase_C	149	133	0.89	4.00E-07	Proteasome	24	23	0.96	0.02
WD40	234	184	0.79	1.00E-06	Cyclin_C	30	28	0.93	0.02
DEAD	114	97	0.85	5.00E-05	HATPase_c	35	30	0.86	0.02
RRM_1	245	171	0.70	0.0003	AAA_5	45	37	0.82	0.02
Kinesin	61	55	0.90	0.001	Mito_carr	59	46	0.78	0.02
PHD	52	47	0.90	0.002	Galactosyl_T	21	20	0.95	0.03
SNF2_N	45	40	0.89	0.005	HA2	21	20	0.95	0.03
PPR_1	301	188	0.62	0.006	Cyclin_N	52	40	0.77	0.03
IQ	56	46	0.82	0.008	Hydrolase	60	45	0.75	0.03
PPR	465	275	0.59	0.008	Miro	114	76	0.67	0.03
AAA	144	98	0.68	0.009	OB_NTP_bind	20	19	0.95	0.04
LSM	26	26	1.00	0.01	Cpn60_TCP1	23	21	0.91	0.04
UCH	45	39	0.87	0.01	KH_1	25	22	0.88	0.04
ResIII	48	40	0.83	0.01	SET	46	35	0.76	0.04
RuvB_N	46	38	0.83	0.01	Histone	67	48	0.72	0.04

### Distinct enrichment of transcription factors in early flower development

**Figure 3:**
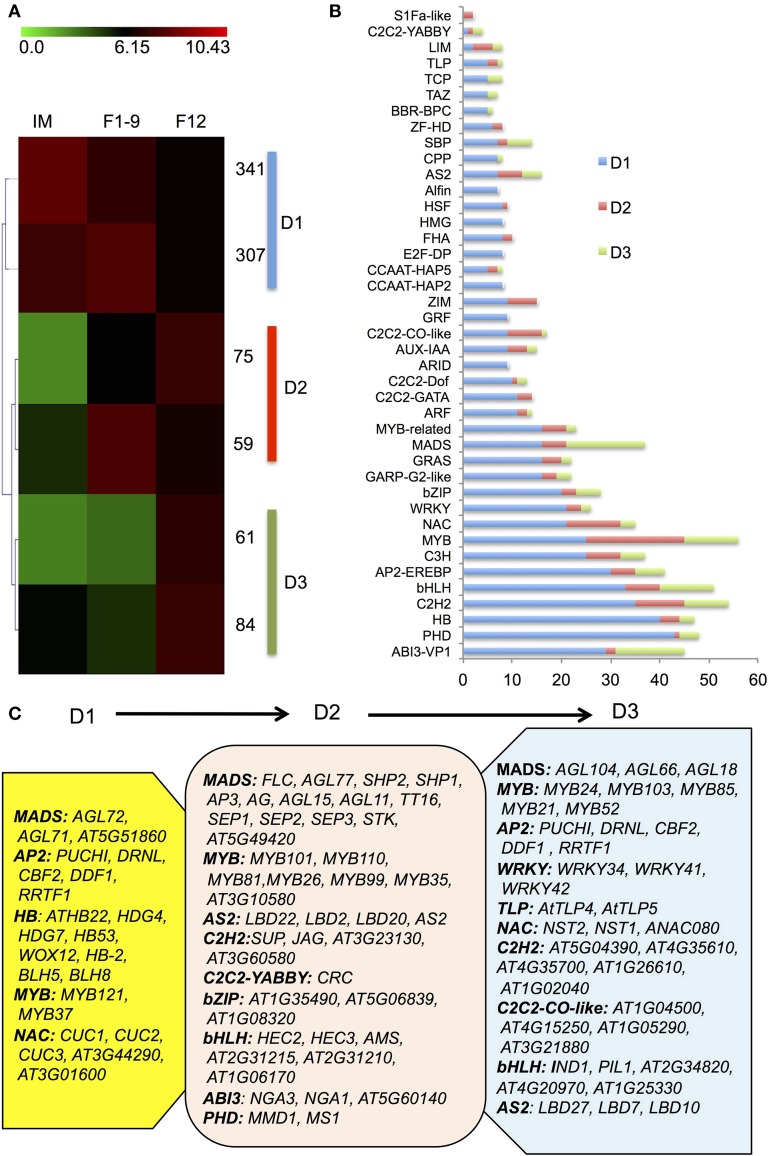
**The transcription factor accumulation profiles. (A)** A heatmap for the transcription factors. 937 significantly differentially expressed transcription factors from IM, F1–9, and F12 were clustered into three groups (D1–D3) using the Self Organization Tree Algorithm. **(B)** Distribution of transcription factor families among D1–D3. **(C)** Representative functions and genes showing different transcription factor families for developmental stage-dependent groups D1 (IM), D2 (F1–9), and D3 (F12).

Identification of transcription factors (TFs) expressed in a specificstage provides a foundation for understanding the transcriptional regulatory networks underlying the development, structure and function of thestage. To investigatethe expressed TFs during flower development, we examined the TFs among IM, F1–9, and F12 and identified a total of 1667 transcription factors, 927 of which showed differential expression compared with the seedling (Figure 3A). Among the 927 TFs, 70% showed highest expression in IM (designated as D1), whereas 14 and 16% showed highest expression in F1–9 (designated as D2) and F12 (designated as D3), respectively (Figure 3B).

D1 mainly contained members of the homeobox domain (HB), MADS, MYB, AP2, and NAC families, suggesting that floral meristem development largely requires those transcription factor (Figure 3B). For example, *homeobox* genes encode transcription factors that contain a classic DNA binding domain with about 60 amino acids and regulate gene expression via Polycomb-dependent modulation of chromatin structure, thereby controlling development in animals, fungi and plants (Zhong and Holland, 2011). Several known members of *HB* (Figure 3C) identified in IM support that early floral development requires active *HB* genes, consistent with the finding that epigenetic reprogramming of gene expression is important for the establishment of initial floral identity (Mukherjee et al., 2009). The co-expressed pattern between *HB* genes and chromatin factors in IM is in agreement with previous studies that a number of floral genes with similar expression patterns and/or associated with each other regulate the expression of downstream genes to ensure proper flower development (Kaufmann et al., 2009, 2010; Deng et al., 2011).

D2 included *MADS-box*, *MYB*, *AS2*, *C2H2*, *bZIP*, *ABI3*, and *bHLH* families (Figure 3C). *MADS-box* genes encode not only key repressors or activators for flowering transition, but also master regulators of reproductive organ identities (Alvarez-Buylla et al., 2010). Our data detected expression of most MADS-box genes known to be involved in flower development (Figure 3C), such as *FLC, SHP1/2, AP3, AG, AGL11/15/77, TT16, SEP1-3, STK, AT5G49420*; *AG*, *AP3*, and *SEP1-3* are genes for the ABCE model, consistent with their known function in floral organ identity (Smaczniak et al., 2012). In addition, genes coding for transcription factors important for microsporogenesis were also uncovered, such as *AMS, MS1, MYB35*, and *MYB99* (Chang et al., 2011), as well as *MMD1* required for meiosis (Yang et al., 2003).

D3 was enriched in *MADS*, *MYB*, *AP2*, *C2H2*, *C2C2-CO-like*, *NAC*, *AUX-IAA-ARF*, and *bHLH* families (Figure 3C). Previous studies showed that auxin-dependent transcriptional regulation requires the auxin/indole-3-acetic acid (Aux/IAA) and auxin response factor (ARF) families of TFs and formation of Aux/IAA-ARFs heterodimers represses auxin signaling (Reed, 2001), which has been demonstrated to participate in pollen development, pollination and fertilization (Sundberg and Ostergaard, 2009), as well as female gametophyte specification (Pagnussat et al., 2009). Indeed, our data identified several known and unknown ARFs and IAAs factors in G3, suggesting that the Aux/IAA-ARF regulatory pathway is vital for late reproductive development. However, the function of other enriched TFs in flowers is still largely unknown. Together, these results demonstrate that flower development at different stages requires common and distinct transcription factor families.

### Identification of specific gene families at distinct stages of flower development

We sought to identify stage-specific genes, which were defined as those genes that were differentially expressed (>4-fold change) at one stage over all other stages studied here using DEG seq. The largest numbers of stage specific genes were identified in the seedling, F12 and meiocytes (1083, 552, and 652 genes, respectively; Table 4). Given the lack of correlation in overall gene expression between the floral transcriptome (F12) and the other stages sampled (Figure 2C), it was not surprising to identify this stage as having the largest number of organ-specific genes. These genes are strong candidates for determining the specific functional components of the nearly mature flower.

**Table 4:** **The specifically expressed genes in one sample compared with others**.

**Fold change**	**Seedling**	**IM**	**F4**	**F1–9**	**F12**	**Meiocyte**
1	2656	1280	686	1740	1632	1636
2	1871	157	118	110	817	1200
4	1083	27	33	26	552	652
8	695	7	16	13	418	424

Interestingly, the F1–9 flower-specific genes with 8-fold changes had 26 genes, including 9 transposons and 5 snoRNAs (Table S4), consistent with the previous finding that transposons and small RNAs were enriched among genes expressed in male meiocytes (Chen et al., 2010; Yang et al., 2011). There are also 12 coding genes, one of which (*AT5G09780*) codes for a transcription factor of the B3 family and two (*AT1G48700* and *AT4G03050*) are for iron binding proteins.

**Figure 4:**
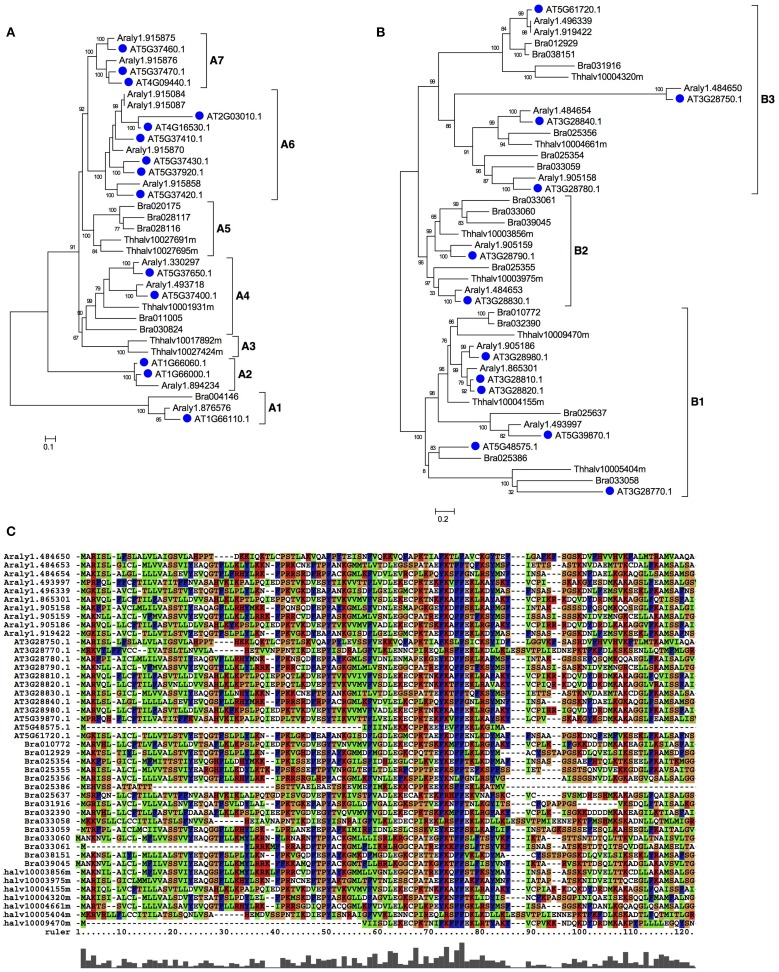
**The phylogenetic tree of *DUF577* and DUF1216 gene families in plant.** The *DUF577* and *DUF1216* gene families were found only in plants, but not in other eukaryotic groups. **(A)** A Maximum likelihood (ML) tree of the *DUF577* gene family using representative species in eudicots and it can be divided into 8 clades, A1~A7. **(B)** An ML tree of *DUF1216* gene family using representative species in eudicots and it can be divided into 3 clades, B1~B3. **(C)** An alignment of the N-terminal regions of DUF1216 proteins. Species names are abbreviated as below: AT-*Arabidopsis thaliana*, Araly-*Arabidopsis lyrata*, Bra-*Brassica rapa*, Thhalv-*Eutrema salsugineuma*.

For meiocyte-specific genes, 424 genes were found with ~8-fold changes and showed enrichment for genes in an insertion of mitochondrial origin on chromosome II, as supported by similar preferential expression in meiocytes reported previously (Chen et al., 2010). The enriched genes also included 45 mitochondrial and 28 chloroplast genes, respectively. Moreover, in addition to previously reported gene families (Yang et al., 2011), we also detected several other enriched gene families, such as *Oxidored_q1*, *Oxidored_q2*, *Oxidored_q3*, *Oxidored_q*4, and *NADHdh*. In particular, most members of the *DUF577* family showed specific expression in meiocytes (Table S5). To further investigate this gene family, we performed phylogenetic analyses of this gene family with members from several representative plant species, including *Arabidopsis lyrata*, *Eutrema salsugineum*, *Brassica rapa*. As shown in Figure 4A, this family can be divided into seven subfamilies, designated as A1-A7. The tree supported that this gene family have experienced expansion and origin in Brassicaceae (Figure 4A). The A6 and A7 subfamilies only included *Arabidopsis lyrata* and *Arabidopsis thaliana*, suggesting an expansion that occurred since the divergence of *Arabidopsis* and other Brassicaceae species. Besides, functions of the *DUF577* and other enriched family genes in meiosis need to be tested.

**Figure 5:**
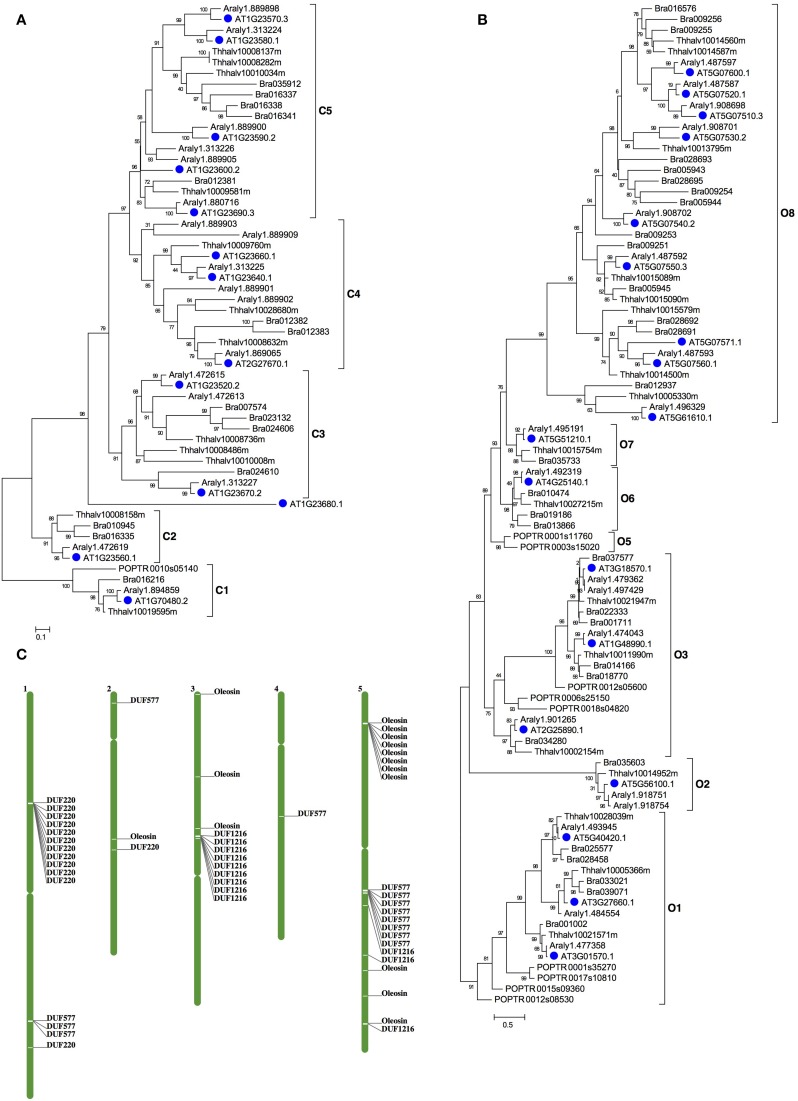
**The phylogenetic tree of *DUF220* and *Oleosin* gene families in plant. (A)** An ML tree of *DUF220* gene family can be divided into 5 clades, C1~C5. **(B)** An ML tree of *Oleosin* gene family can be divided into 8 clades, O1~O8. Species names are abbreviated as below: AT-*Arabidopsis thaliana*, Araly-*Arabidopsis lyrata*, Bra-*Brassica rapa*, Thhalv-*Eutrema salsugineum*; POPTR-*Populus trichocarpa*. **(C)** The chromosomal positions of the *Arabidopsis DUF577*, *DUF1216*, *DUF22*0, and *Oleosin* genes. The names of genes refer to locus ID as listed in Table S5.

Similarly for F12, the enriched families included *DUF121*6, *Oleosin* and *DUF220*. Most members of the three gene families had specific expression in F12; these gene families contain lineages that originated and expanded within Brassicaceae (Table S5 and Figures 4, 5). Phylogenetic analyses of the *DUF1216* family suggested that this gene family is specific to Brassicaceae, without homologs in other plants, and experienced gene duplication during Brassicaceae history (Figure 4B). Interestingly, the N-terminal region of DUF1216 proteins had putative signal peptides with similar sequences, according to the SignalP prediction (http://www.cbs.dtu.dk/services/SignalP/). The predicted signal peptide contains a large number of hydrophobic amino acids, a conserved basic amino acid and a conserved cysteine at the ninth position (Figure 4C). *At5g07750* of the *Oleosin* family was reported to have experienced positive selection (Schein et al., 2004). However, expression of each of three tandem duplicated genes in the Oleosin family (*AT5G07510*: 10081.76, *AT5G07550*: 29536.45, *AT5G07560*: 10942.38) had extraordinarily high levels of more than RPKM of 10, 000, suggesting that such high expression levels are important for F12 for later functions. Analysis of the *Arabidopsis* genome indicates that tandem duplication contributed to the expansion of *DUF1216* in Brassicaceae, as well as the expansion of the *Oleosin*, *DUF220* and *DUF577* families (Figure 5C). This pattern is different to those of *SET*, *JmjC*, and *Rhomboid* gene families, which are more likely to be retained after whole genome duplication events (Zhou and Ma, 2008; Zhang and Ma, 2012; Li et al., 2014).

## Conclusions

The analysis of *Arabidopsis* floral transcriptome datasets presented here provides a valuable resource of candidate genes for further studies to understand the flower development program. We provided evidence for at least 23,961 genes that are expressed in the *Arabidopsis* flower. Compared with seedling, over 10,000 DEGs were identified, revealing novel and different molecular characteristics in the developing flower such as regulatory genes, genes for high-energy production, and transposable elements. These results showed that flower development at different stages requires common and distinct transcription factor families. The gene expression in F12 was dramatically different from that for early flower development (F1–9, F4, and IM).

In addition to identifying floral developmental gene candidates, we found many genes or gene families specifically expressed at one stage. Many transposable element genes, at least 45 mitochondrial and 28 chloroplast genes showed specific expression in meiocytes. The *SCRL*, *SLR1-BP*, *DUF1216*, *Oleosin*, and *DUF220* gene families showed specific expression in F12 and *DUF577* genes were detected to have specific expression meiosis. These specifically expressed genes have functions that are closely related to reproductive development, showed that mature flowers require many more specifically or differentially expressed genes than early flowers. These gene families expanded dramatically within the Brassicaceae lineage, suggesting novel functions that are possibly important for the origin and evolution of Brassicaceae. This dataset can be useful for discovering functional genes at different stages of the flower development and provide clues for the molecular and regulatory relationships between different stages.

### Conflict of interest statement

The authors declare that the research was conducted in the absence of any commercial or financial relationships that could be construed as a potential conflict of interest.

## Supplementary material

Figure S1 **Biological replicates were highly reproducible**.

Figure S2 **Comparison between transcriptomes from RNA-Seq and microarray**.

Table S1 **The expression of all genes in six samples**.

Table S2 **The 126 known *Arabidopsis* flower development genes**.

Table S3 **The expression of *SCRL* and *SLR1-BP* genes in different samples**.

Table S4 **The expression of the 26 specific genes in F1–9**.

Table S5 **The expression of *DUF577*, *DUF1216*, *DUF220* and *Oleosin* genes in different samples**.
