# Supplementary figures and images for: Analysis of Arabidopsis floral transcriptome: detection of new florally expressed genes and expansion of Brassicaceae-specific gene families
Source: Front Plant Sci. 2015 Jan 20;5:802. doi: 10.3389/fpls.2014.00802 (PMC4299442; doi:10.3389/fpls.2014.00802)

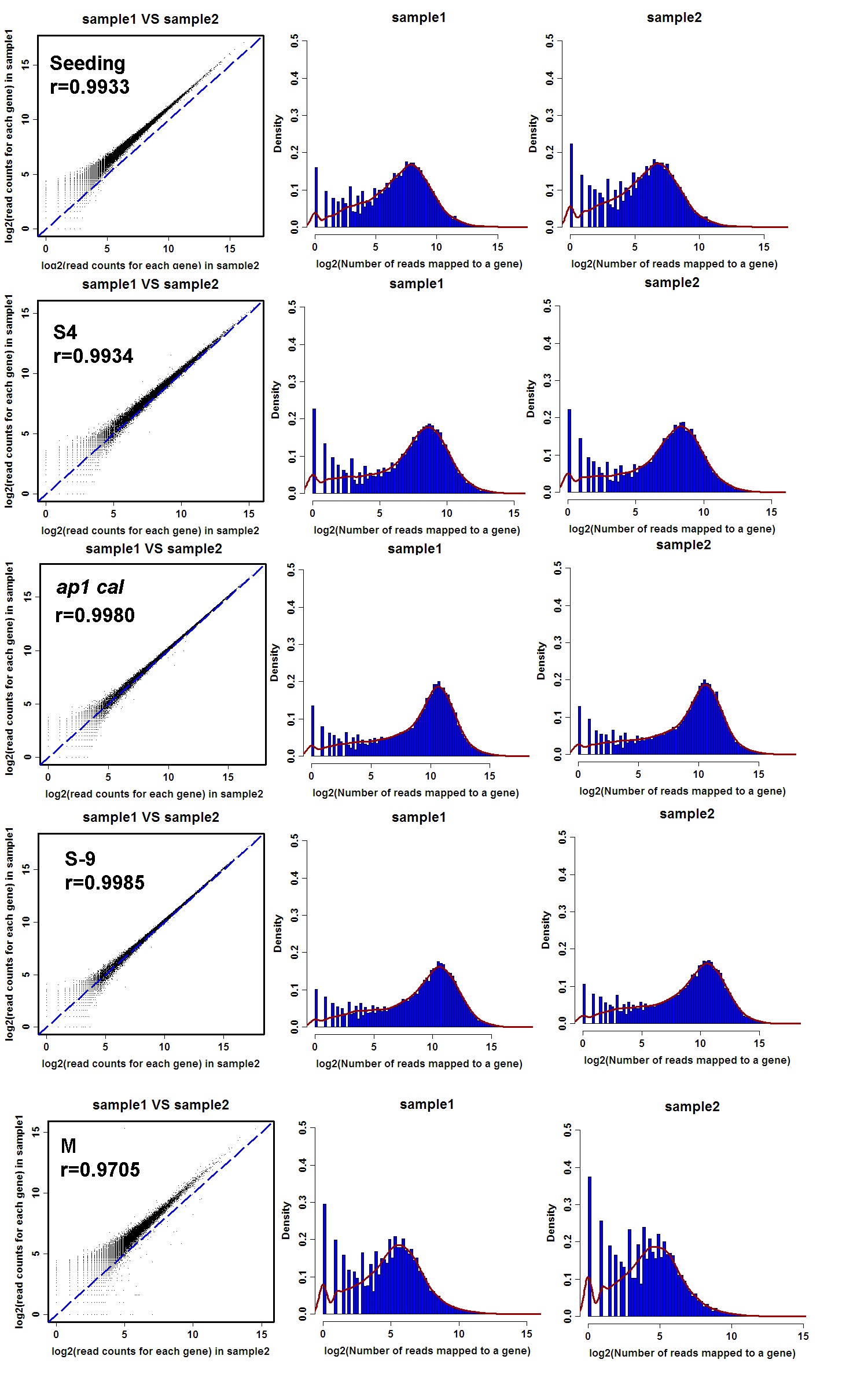

Supplement: Figure S1 — Biological replicates were highly reproducible. [file Image1.JPEG]

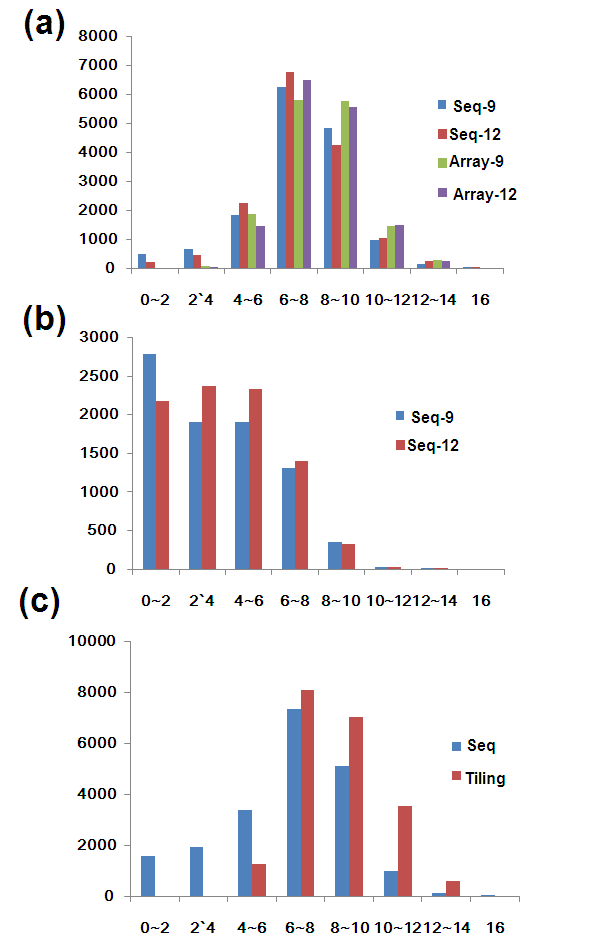

Supplement: Figure S2 — Comparison between transcriptomes from RNA-Seq and microarray. [file Image2.TIF]
